# Persistent but dysfunctional mucosal SARS-CoV-2-specific IgA and low lung IL-1β associate with COVID-19 fatal outcome: A cross-sectional analysis

**DOI:** 10.3389/fimmu.2022.842468

**Published:** 2022-09-29

**Authors:** Maria Julia Ruiz, Gabriel Siracusano, Andréa Cottignies-Calamarte, Daniela Tudor, Fernando Real, Aiwei Zhu, Claudia Pastori, Claude Capron, Arielle R. Rosenberg, Nigel Temperton, Diego Cantoni, Hanqing Liao, Nicola Ternette, Pierre Moine, Mathieu Godement, Guillaume Geri, Jean-Daniel Chiche, Djillali Annane, Elisabeth Cramer Bordé, Lucia Lopalco, Morgane Bomsel

**Affiliations:** ^1^ Mucosal Entry of HIV and Mucosal Immunity, Institut Cochin, Paris-Descartes University, Paris, France; ^2^ INSERM U1016, Paris, France; ^3^ CNRS UMR8104, Paris, France; ^4^ Immunobiology of HIV Unit, San Raffaele Scientific Institute, Milan, Italy; ^5^ AP-HP, Hôpital Ambroise Paré, Service d'Hématologie, Boulogne-Billancourt, France; ^6^ AP-HP, Hôpital Cochin, Service de Virologie, Paris, France; ^7^ Viral Pseudotype Unit, Medway School of Pharmacy, The Universities of Kent and Greenwich at Medway, Chatham, United Kingdom; ^8^ Centre for Cellular and Molecular Physiology, Nuffield Department of Medicine, University of Oxford, Oxford, United Kingdom; ^9^ FHU SEPSIS (Saclay and Paris Seine Nord Endeavour to PerSonalize Interventions for Sepsis), RHU RECORDS (Rapid rEcognition of CORticosteroiD resistant or sensitive Sepsis), Department of Intensive Care, Hôpital Raymond Poincaré (APHP), Laboratory of Infection and Inflammation – U1173, School of Medicine Simone Veil, University Versailles Saint Quentin – University Paris Saclay, INSERM, Garches, France; ^10^ AP-HP, Hôpital Ambroise Paré, Service de Réanimation, Boulogne-Billancourt, France; ^11^ Université de Versailles-St Quentin en Yvelines, Versailles, France; ^12^ AP-HP, Hôpital Cochin, Service de Réanimation, Paris, France

**Keywords:** SARS-CoV-2, COVID-19, mucosal immunity, IgA, severe infection, inflammatory cytokine

## Abstract

The role of the mucosal pulmonary antibody response in coronavirus disease 2019 (COVID-19) outcome remains unclear. Here, we found that in bronchoalveolar lavage (BAL) samples from 48 patients with severe COVID-19-infected with the ancestral Wuhan virus, mucosal IgG and IgA specific for S1, receptor-binding domain (RBD), S2, and nucleocapsid protein (NP) emerged in BAL containing viruses early in infection and persist after virus elimination, with more IgA than IgG for all antigens tested. Furthermore, spike-IgA and spike-IgG immune complexes were detected in BAL, especially when the lung virus has been cleared. BAL IgG and IgA recognized the four main RBD variants. BAL neutralizing titers were higher early in COVID-19 when virus replicates in the lung than later in infection after viral clearance. Patients with fatal COVID-19, in contrast to survivors, developed higher levels of mucosal spike-specific IgA than IgG but lost neutralizing activities over time and had reduced IL-1β in the lung. Altogether, mucosal spike and NP-specific IgG and S1-specific IgA persisting after lung severe acute respiratory syndrome coronavirus 2 (SARS-CoV-2) clearance and low pulmonary IL-1β correlate with COVID-19 fatal outcome. Thus, mucosal SARS-CoV-2-specific antibodies may have adverse functions in addition to protective neutralization.

**Highlights:**

Mucosal pulmonary antibody response in COVID-19 outcome remains unclear. We show that in severe COVID-19 patients, mucosal pulmonary non-neutralizing SARS-CoV-2 IgA persit after viral clearance in the lung. Furthermore, low lung IL-1β correlate with fatal COVID-19. Altogether, mucosal IgA may exert harmful functions beside protective neutralization.

## Introduction

The new pandemic coronavirus disease 2019 (COVID-19) is a highly transmittable mucosal viral infection. It is caused by the severe acute respiratory syndrome coronavirus 2 (SARS-CoV-2) (1), an enveloped positive-strand RNA virus (2). SARS-CoV-2 infection commonly induces fever, unproductive cough, myalgia, and fatigue and, in extreme cases, leads to the development of acute respiratory distress syndrome (ARDS) and progression from ARDS to death (3).

The SARS-CoV-2 viral membrane contains the spike (S), a viral glycoprotein essential for virus entry in target cells. The S protein is composed of two subunits, S1 and S2, which are cleaved by a serine-like protease (4). S1 contains the receptor-binding domain (RBD) that binds to the host cell receptor angiotensin-converting enzyme 2 (ACE2). The cleavage releases the S2 domain, which, in turn, mediates viral fusion in an endosomal compartment (4) as in other coronaviruses (5), resulting in cell infection and virus replication.

Given the tremendous effort made by the scientific community, 24 vaccines have been approved for use in humans as of July 2022 (6). While these findings are more than encouraging, the course that the pandemic will take due to vaccination can only be assessed in the long term. In addition, new epidemic waves arise due to the emergence of SARS-CoV-2 variants (7). Therefore, investigations on SARS-CoV-2 pathophysiology remain a priority, especially in the respiratory tract, the main portal of entry and replication site of the virus.

The humoral immune response against SARS-CoV-2 has been extensively evaluated in the serum of COVID-19 individuals (8). Seroconversion occurs between 7 and 14 days after the onset of symptoms in the majority of subjects. Antibody titers persist for weeks following virus clearance (9), and the neutralizing activity is detectable within a week after the onset of symptoms (3, 10).

Conversely, very limited data exist on the mucosal SARS-CoV-2-specific immune response, especially in the respiratory tract, the main portal of entry and replication site of the virus. Bronchoalveolar lavage (BAL) fluid is representative of the pulmonary microenvironment in terms of lung cell types, cytokines, and mucosal antibodies. BAL appears thus as an accessible fluid ideal for profiling the mucosal antibody response to SARS-CoV-2 during the course of the infection. IgA is the predominant antibody in mucosal regions, such as the respiratory tract (11) and the second most abundant after IgG in serum. The protective role of secretory IgA during COVID-19 was highlighted in different studies, most of them performed at a systemic level (12, 13). Sterlin et al. (13) measured the frequency of antibody-secreting cells and the presence of SARS-CoV-2-specific neutralizing antibodies in the serum, saliva, and BAL and found that the humoral response was dominated by IgA. Peripheral expansion of IgA plasmablasts with mucosal homing potential was detected shortly after the onset of symptoms. Serum IgA contribution to virus neutralization was higher than that of IgG, but spike-specific serum IgA decreased notably 1 month after the onset of symptoms. In contrast, saliva IgA remained detectable for up to 11 weeks post-infection (13). Which factors contributed to severe COVID-19 mucosal IgA in the lung remains unclear.

While serum monomeric IgA is produced by plasma cells in the bone marrow, secretory IgA is produced locally as dimeric IgA by plasma cells residing at the mucosal surfaces. Consequently, systemic and humoral immune responses are highly compartmentalized, and the systemic and mucosal humoral immune responses have different repertoires (14). A protective anti-SARS-CoV-2 IgA response in the lung may block infection and in turn transmission and is highly desirable for designing future protective vaccines (15–17). However, IgA may also play harmful roles in SARS-CoV-2 pathogenesis (18). Mucosal antibodies raised during infection may contribute to the protective hybrid immunity resulting from vaccination after COVID-19 recovery that appears superior compared with vaccination of SARS-CoV-2 naïve individuals or immunity raised by natural SARS-CoV-2 infection (19).

Memory B cells can expand and differentiate into antibody-secreting cells upon an antigenic challenge (20). The generation of memory B cells in the blood in the context of SARS-CoV-2 infection has been recently evaluated in several sophisticated studies (19, 21). Surprisingly, there is no evaluation of B cells in the lungs. In this context, BAL represents a valuable tool to explore this field in severe SARS-CoV-2-infected subjects.

A specific cytokine pattern has been shown to contribute to COVID-19 severity with the development of a cytokine storm syndrome accompanied by a hyperinflammation syndrome (22). The serum cytokine profile detected in COVID-19 severe cases includes increased production of IL-2, IL-7, granulocyte–macrophage colony-stimulating factor (GM-CSF), TNF-alpha, CXCL10, MCP1/CCL2, and MIP1-alpha (22). Hyperinflammation driven by SARS-CoV-2 infection is thus strongly correlated with COVID-19 mortality. The cytokine patterns at the local lung level and their contribution to COVID-19 have been recently reviewed elsewhere (23), but their correlation with mucosal IgG or IgA remains unclear. Therapeutic strategies for counteracting inflammation in COVID-19 severe cases are required for improving patient recovery from respiratory failure. To achieve this goal, it is mandatory to clarify the cytokine profile not only in serum but also in the lung.

In this study, we profiled the mucosal-specific IgA and IgG and their corresponding B cells in patients with severe COVID-19 stratified in two categories, either experiencing virus replication in the lung and after the virus has been cleared from the lung. The neutralizing activities of these antibodies were evaluated as well as the mucosal cytokine profile in BAL. Correlations between these parameters and patient clinical outcomes reveal a signature associated with non survival.

## Methods

### Patients and ethical statements

This non-interventional study was approved by the institutional review board of the ethical committee for research (CER) of the University of Paris Saclay (CER-Paris-Saclay-2020-050) and conformed to the principles outlined in the Declaration of Helsinki. Accordingly, all participants were informed in writing about the study and were given the option to not participate. We studied prospective samples from 48 COVID-19 and 21 non-COVID patients admitted at the Cochin (Paris, France), Ambroise Paré (Boulogne-Billancourt, France), and Raymond Poincaré (Garches, France) Hospitals between March and June 2020. All patients had a COVID-19 diagnosis confirmed by SARS-CoV-2 RNA RT-qPCR in nasopharyngeal swabs at the hospital. Clinical data submitted by the participating centers were anonymized and encrypted.

### Sample collection

BAL samples were collected as described (24) and processed as indicated in our recent study (25). Briefly, a volume of 50 ml of isotonic saline was injected with a recovery of 6 to 18 ml, and the collected fluid was processed within 3 h. BAL was passed through a 70 μm strainer and collected in a 50 ml tube. After the centrifugation of 500 g for 10 min, fluid was collected, aliquoted at 1 ml, and stored until use at −80°C in a biosafety level 3 (BSL3) laboratory. The BAL cells were resuspended in 10% dimethyl sulfoxide (DMSO) in fetal calf serum (FCS) and stored at −80°C until use in flow cytometry analyses. BAL fluids without cells were aliquoted in 60 μl fractions, inactivated at 56°C for 30 min in the BSL3 facility, and stored at −80°C for subsequent use.

### Enzyme-linked immunosorbent assays

The concentration of total IgG and IgA in BAL secretions was measured by sandwich enzyme-linked immunosorbent assay (ELISA) as we described (26) using polyclonal goat antihuman IgG or IgA (Biosystems, Burlingame, CA, USA) for coating and polyclonal goat anti-human IgG (Nordic, Tilburg, The Netherlands) or polyclonal goat anti-human IgA (Nordic) for detection; standards were purified human serum IgG (Sigma, St. Louis, MI, USA; I2511) or purified human colostral IgA (Sigma, I2636). BAL IgG and IgA specific to SARS-CoV-2 S1, S2, RBD, and nucleocapsid protein (NP) were determined by an ELISA as described below. IgG and IgA anti-S1 detection was performed using the anti-SARS-CoV-2 ELISA Kit (EI 2606-9620 G and EI 2606-9620 A; Euroimmun, Mountain Lakes, NJ, USA) following the manufacturer’s instructions. IgG and IgA to NP were measured using the NOVATEC ELISA KIT according to the manufacturer’s instructions. For IgG and IgA anti-S2 and anti-RBD quantification, 96-well, flat-bottomed plates (Nunc-Immun Microwell, Thermo Fisher Scientific, Odense C, Denmark) were coated overnight at 4°C with 1 ng/well, or 100 ng/well, of SARS-CoV-2 spike S2 protein and SARS-CoV-2 spike RBD Wuhan protein (LifeTein, Somerset, NJ, USA) and recombinant human SARS-CoV-2 spike RBD variants Alpha (B.1.1.7), Beta (B.1.351), P.1 (Gamma), and Delta (B.1.617) (Diaclone, Besançon, France). After 24 h, a blocking solution (200 µl per well of bovine serum albumin (BSA) 2% diluted in phosphate-buffered saline (PBS) with 0.1% Tween 20 (PBST)) was added, and plates were incubated for 2 h at 37°C followed by five washes with PBST. BAL samples were diluted in PBST (1/50 and 1/100 dilution), and 100 µl of diluted samples were added to the plates for 2 h at 37°C. After several washes, goat anti-human IgG labeled with horseradish peroxidase (HRP) or goat anti-human IgA-HRP (Jackson Immunoresearch, Ely, UK) was added to each well for 1 h at room temperature. The reaction was developed with tetramethylbenzidine (TMB)-ELISA solution (Eurobio Scientific, Essonnes, Ile-de-France, France) for 15 min prior to stopping with H_3_PO_4_ (1 M). The absorption at 450 nM (OD450) was read on a Spectramax spectrophotometer (Molecular Devices, Wokingham, UK). Samples from COVID-19-negative subjects in the intensive care unit (ICU) were tested as negative controls. As for another mucosal sampling, the volume of mucosal BAL sampled in each individual varies from individual to individual for various reasons such as patient morphology and COVID-19 pathology specificities (27). Thus, to compare antigen-specific antibody isotype, IgG, and IgA, in the BAL, for each isotype, we normalized the OD450 values measuring specific binding to each antigen to the total antibody isotype concentration as we described earlier (26, 28, 29). Results are shown in arbitrary units (AU) calculated as follows: (OD450 measured in antigen-specific IgA or IgG ELISA/total IgA or IgG concentration (μg/ml)).

As internal standard control, the WHO International Standard (WHO IS, National Institute for Biological Standards and Control, NIBSC, UK, cod. 20/136) and the WHO Reference Panel (WHO RP, NIBSC, cod. 20/268) for anti-SARS-CoV-2 antibody were tested at 1:100 dilution in the ELISA for S1 and S2 and the Novatec kit for NP to check the concordance with our results, as previously described (30).

For the detection of IgA-SARS-CoV-2 immune complexes, ELISA was performed as above, except for the coating conditions. Plates were coated with the polyclonal rabbit anti-SARS-CoV-2 spike (Genetex GTX135356) at 50 ng/well. Specificity was established using BAL from three different non-COVID individuals. These values were considered as background and subtracted from the presented data.

### Cell lines

HEK 293T/17 cells were obtained from the American Type Culture Collection (ATCC, Manassas, VA, USA) and cultured in Dulbecco’s modified Eagle medium (Lonza, Basel, Switzerland) supplemented with 10% fetal bovine serum, 100 units/ml of penicillin, and 100 µg/ml of streptomycin (Euroclone, Pero, Italy). HEK 293T/17-ACE2/TMPRSS2 cells were generated by co-transfection of pCAGGS encoded human ACE2 and human TMPRSS2 using FuGENE^®^ HD Transfection Reagent (Promega, Madison, WI, USA) according to the manufacturer’s instruction. After 24 h, cells were detached and used for downstream assays.

### Production of SARS-CoV-2 pseudotyped viruses

A lentivirus-based SARS-CoV-2 pseudotyped virus (PSV) was generated, as previously described (31). Briefly, HEK 293T/17 cells were co-transfected with a SARS-CoV-2 spike encoding plasmid, a p8.91 HIV Gag-pol packaging construct, and a pCSFLW plasmid encoding a firefly luciferase reporter using Fugene^®^ HD transfection reagent, according to the manufacturer’s instruction. To generate PSVs of the SARS-CoV-2 variants of concern, spike plasmids encoding the mutations for Alpha (B.1.1.7), Beta (B.1.351), and Gamma (P.1) were commercially synthesized and used instead. Cells were incubated for 48 h prior to collecting and filtrating supernatant containing PSVs, using a 0.45 µm cellulose acetate filter. PSVs were then aliquoted and stored at −80°C.

### Titration of SARS-CoV-2 pseudotyped viruses

Viral titers were determined by transducing 10^4^ HEK 293T/17-ACE2/TMPRSS2 cells with twofold serial dilutions of PSVs to each well of a 96-well titration plate, as previously described (31) After 48 h post-incubation at 37°C 5% CO_2_, firefly luciferase expression was quantified by the Bright-Glo™ assay luciferase system (Promega) and the VICTOR X Light Luminescence Plate Reader (PerkinElmer, Waltham, MA, USA). Each relative luminescence unit (RLU) value obtained at different PSV dilution points was converted into RLU/ml, and the arithmetic mean of these concentrations was considered as the PSV production titer (expressed as RLU/ml).

### Pseudotype-based microneutralization assay

Neutralization activity of previously heat-inactivated (56°C for 30 min) plasma from COVID-19 patients was measured using a single round PSV infection of HEK 293T/17-ACE2/TMPRSS2-transfected cells. Plasma collected prior to the emergence of SARS-CoV-2 was used as negative controls. Neutralization assays were performed by incubating 10^6^ RLU of SARS-CoV-2 Wuhan, Alpha, Beta, or Gamma pseudotyped viruses with endpoint twofold serial dilutions of BAL samples (starting from 1:5) at 37°C 5% CO_2_ for 1 h before addition of 10^4^ HEK 293T/17-ACE2/TMPRSS2 cells per well. All samples were measured twice in duplicate. After 48 h at 37°C, the cells were lysed, and luciferase activity was measured as previously reported (32). Neutralization titers were converted into half-maximal inhibitory concentration (IC50) using Prism software, as previously described (32).

### Flow cytometry

BAL cells were thawed from frozen aliquots and fixed with 4% paraformaldehyde (PFA) for 30 min. After successive washes with PBS-BSA, cells were incubated for 30 min, at room temperature, with the following antibodies coupled to fluorophores diluted in permeabilization buffer (PBS 0.1% Saponin 2% FCS): CD3 Pacific Blue (BD ref: 558117, 1:20 v/v), CD19 APC-H7 (BD ref: 560177, 1:40 v/v), CD27 PE (BD ref: 566944, 1:20 v/v), CD21 PE-Cy7 (BD ref: 561374, 1:20 v/v), CD38 BV711 (BD ref: 563965, 1:40 v/v), and CD138 APC (BD ref: 347216, 1:20 v/v). Then, cells were labeled with either human fluorescein isothiocyanate (FITC)-conjugated IgA (Jackson ref: 309-095-011, 1:100 v/v) or human FITC-conjugated IgG (Jackson ref: 709-096-149, 1:50 v/v). Cells were then analyzed by flow cytometry (Guava easyCyte 12HT base system, Millipore, Billerica, MA, USA) using a gating strategy shown in **Supplementary Figure 1** to evaluate various B-cell subset frequencies.

### Cytokine analyses

After frozen aliquots of BAL fluid were thawed, samples were directly processed for multiplex detection of the following cytokines, according to the distributor’s instructions: MIP-1α, G-CSF, M-CSF, IL-1α, IL-1β, IL-6, IL-8, TNF-α S100A8, S100B, and CXCL4 (R&D Luminex, R&D, Austin, TX, USA). Samples were analyzed in a Bio-Plex 200 system (Bio-Rad, Hercules, CA, USA) following the manufacturer’s instructions.

### Statistical analysis

Analysis of data was performed using Microsoft^®^ Excel 2011 and GraphPad Prism^®^ version 9 (GraphPad software). Summary statistics, mean with standard error of the mean (SEM) and percentages, are shown. Statistical tests were performed considering non-normal distributions (non-parametric tests, unpaired Mann–Whitney test, or paired Wilcoxon test, as indicated). Correlations were assessed by two-tailed Spearman’s correlation coefficients. All tests were two-sided with *p*-values of 0.05 or less denoting statistical significance. The results are presented as box or violin plots with individual values represented as dots.

Univariate and multivariate logistic regression models were used to assess predictors of hospital mortality, with odds ratio (OR) and 95% confidence intervals [95% CIs] used as the measure of association with the outcome.

Bayesian logistic regression was applied to BAL cytokine measurements using the RStanArm package in the R language.

## Results

### The presence of SARS-CoV-2 in bronchoalveolar lavages from individuals with severe COVID-19 defines an active virus replication phase in the nasopharyngeal mucosa

**Figure 1:**
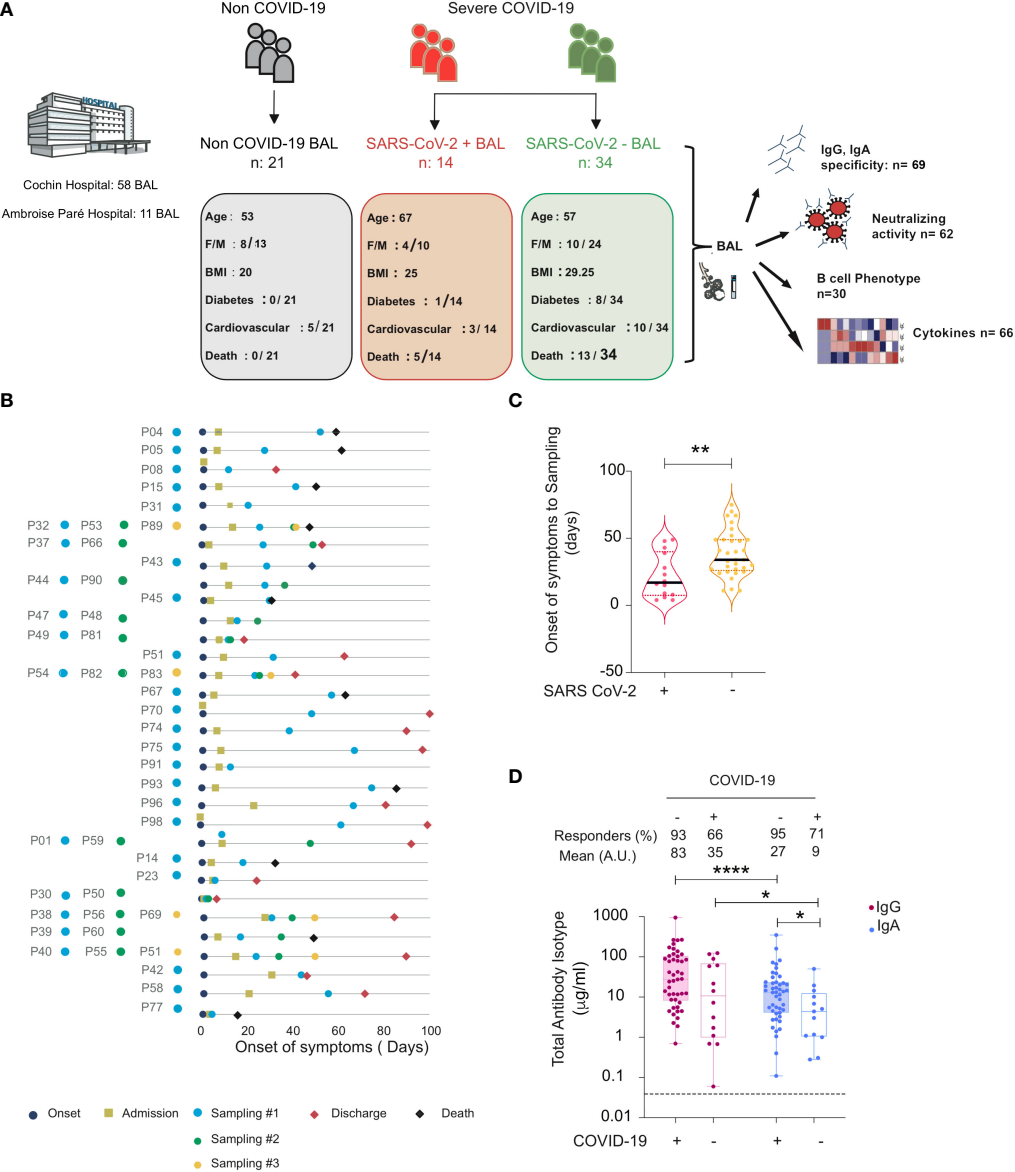
Total and specific IgG and IgA in BAL from SARS-CoV-2-infected and non-infected individuals. **(A)** Graphical representation showing the overall study design and the characteristics (number, age, body mass index (BMI), sex (F = female, M = male), diabetes, and fatality rates) of the individuals included in the study. Illustration with images from Servier Medical Art, licensed under the Creative Commons Attribution 3.0 Unported License. **(B)** Timeline of the course of disease for enrolled patients infected with SARS-CoV-2. **(C)** SARS-CoV-2 containing (SARS-CoV-2+) samples correspond to the early phase, whereas those lacking virus (SARS-CoV-2−) correspond to a late phase of the disease. Violin plots of time from onset of symptoms to sampling for each sample in SARS-CoV-2+ and SARS-CoV-2− BAL. *p*-Values were calculated by using Mann–Whitney test. **(D)** Comparison between values of total IgG and IgA (μg/ml) in BAL from SARS-CoV-2-infected individuals (SARS-CoV-2+ and SARS-CoV-2− BAL) and COVID-19 non-infected individuals. *p*-Values were calculated by using Wilcoxon test: *, *p* < 0.05; **, *p* < 0.01; ****, *p* < 0.0001. Dashed line: cutoff value for antibody detection. Negative values are not shown. BAL for bronchoalveolar lavage.

A total of 69 BAL samples were collected from SARS-CoV-2-infected (severe COVID-19, n = 34) and non-infected (non-COVID-19, n = 21) individuals in the intensive care unit between March and June 2020 whose clinical data are summarized in **Table 1**. Samples were obtained at the enrolment, and 10 individuals provided longitudinal samples. Furthermore, BAL samples were stratified in two groups according to viral gene detection, referred to as SARS-CoV-2+ BAL (mean viral load in BAL: 3.27 × 10^6^ ORF1 copies/ml ± 2.11 × 10^6^), and RT-qPCR negative, referred to as SARS-CoV-2 neg BAL (no detectable viral load in BAL). The experimental design and the cross-sectional sampling during the disease course of the patients are shown in **Figures 1A, B** respectively.

**Table 1:** Clinical data of individuals involved in the study.

	BAL SARS-CoV-2+ N = 11	BAL SARS-CoV-2 neg N = 23	Non COVID-19− N = 21	Total N = 55
	Mean (IQR)	Mean (IQR)	Mean (IQR)	Mean (IQR)
Age (years)	65 (69–76)	64.5 (58–72)	60 (45–72)	63 (48–73)
	N (%)	N (%)	N (%)	N (%)
Gender Female Male	3 (27) 8 (73)	7 (30) 16 (70)	8 (38) 13 (62)	18 (32) 37 (68)
Outcome Survivor Non-survivor	7 (64) 4 (36)	14 (61) 9 (39)	21 (100) 0 (0)	42 (76) 13 (24)
Reason of admission Pneumonia/sepsis/ARDS Fever/cough Dyspnea Hypercapnic coma–pneumonia Sarcoidosis Bronchial congestion Psychomotor slowness Anosmia and ageusia Left adrenal mass N/A	3 (27) 4 (36) 3 (27) 1 (9) 0 (0) 0 (0) 0 (0) 0 (0) 0 (0) 0 (0)	8 (34) 5 (21) 8 (34) 0 (0) 0 (0) 0 (0) 0 (0) 0 (0) 0 (0) 2 (8)	0 (0) 6 (28) 3 (14) 0 (0) 1 (4) 2 (9) 1 (4) 1 (4) 2 (9) 5 (23)	11 (20) 15 (27) 14 (25) 1 (1) 1 (1) 2 (3) 1 (1) 1 (1) 2 (3) 7 (12)
Diabetes Yes No N/A	1 (9) 9 (81) 1 (9)	14 (60) 6 (26) 3 (13)	0 (0) 17 (80) 4 (19)	15 (27) 32 (58) 8 (14)
Obesity Yes No N/A	1 (9) 9 (81) 1 (9)	7 (30) 15 (65) 1 (4)	2 (9) 18 (85) 1 (7)	10 (18) 42 (76) 3 (5)
Cardiovascular disease Yes No N/A	2 (18) 8 (72) 1 (9)	5 (21) 17 (74) 1 (4)	4 (19) 13 (62) 4 (19)	11 (20) 38 (69) 6 (11)

BAL, bronchoalveolar lavage; IQR, interquartile range; ARDS, acute respiratory distress syndrome. N/A, Non available.

When the presence of SARS-CoV-2 in BAL was correlated to patient clinical data, the mean time from the onset of symptoms to sampling was shorter for virus-containing BAL (22 ± 4.4 mean days) compared to virus-free BAL (38 ± 3 mean days, *p* = 0.003, unpaired Mann–Whitney test, **Figure 1C**). Although they partially overlapped due to the heterogeneous dynamic of viral persistence (33, 34), the two groups appeared thus to be statistically significantly different when stratified by time from onset of symptoms. We therefore associated the presence of the virus in BAL with the phase of the disease (35). SARS-CoV-2+ BAL corresponded to an early phase of virus replication, whereas SARS-CoV-2 neg BAL corresponded to a late phase of the infection when after the virus has been cleared from the lung. Accordingly, SARS-CoV-2+ BAL and SARS-CoV-2 neg BAL are referred to as BAL from the early and late phases of COVID-19 disease, respectively.

### Bronchoalveolar lavages from individuals with severe COVID-19 are a suitable fluid to investigate the presence of SARS-CoV-2-specific IgG and IgA antibodies

To study the dynamics of the lung humoral anti-SARS-CoV-2 immune response in severe COVID-19, we first quantified the presence of total IgG and IgA in BAL samples by ELISA (**Figure 1D**). Although monomeric IgA can be present in BAL, the prevalent class of antibodies in BAL is mucosal secretory IgA (13). We thus refer in the following to IgA as mucosal IgA. Total IgG and IgA were detected in >90% of early COVID-19 samples, whereas the proportion decreased to >65% in late COVID-19 samples when the virus was undetectable. The concentration of total IgG was statistically higher than that of IgA in all BAL samples from COVID-19 patients (early COVID-19 sample mean 82 ± 22 μg/ml for IgG *vs* 27 ± 8.1 μg/ml for IgA, *p* < 0.0001, late COVID-19 sample mean: 27 ± 8.1 μg/ml for IgG *vs* 9 ± 3.8 μg/ml for IgA, *p* = 0.01).

### Spike- and N-specific IgA and IgG mucosal responses develop when the virus replicates in bronchoalveolar lavages and persist after virus elimination with more abundant specific IgA than IgG

S1, which includes the ACE2 RBD, and S2 subunits, accessible at the virus surface, are likely targets for COVID-19 protective antibodies. The internal NP, the most abundant in infected cells, offers a sensitive marker of infection (36). IgG and IgA targeting these antigens were quantified in all BAL samples from severe COVID-19 patients.

**Figure 2:**
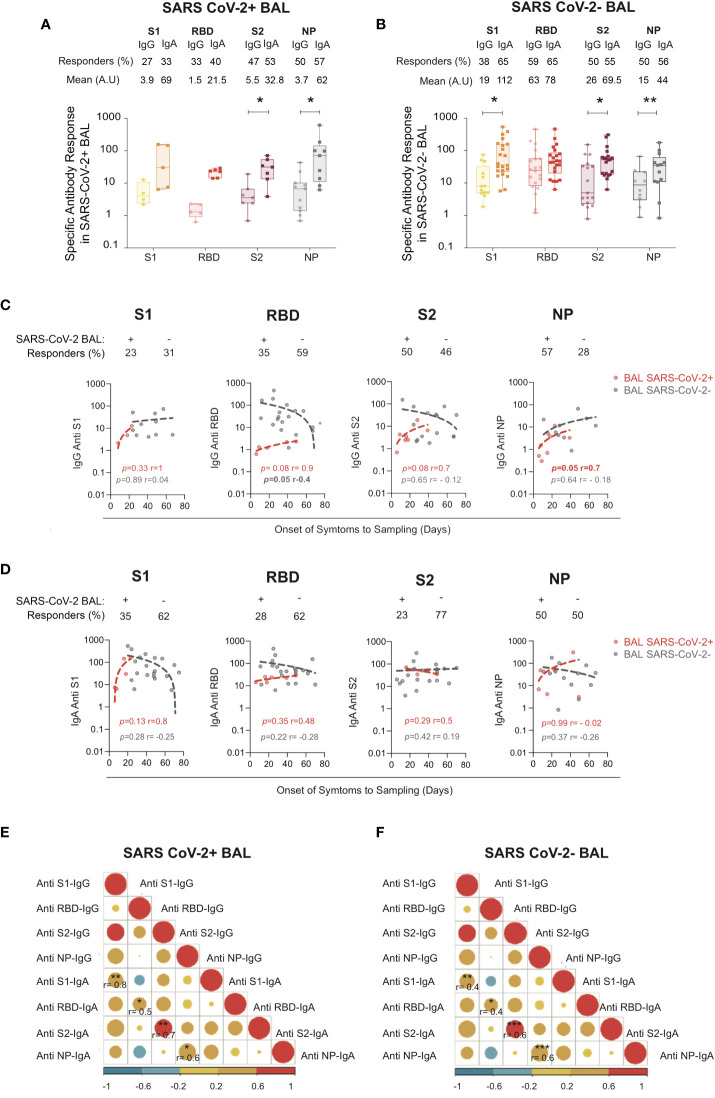
S1-, RBD-, S2-, and NP-specific IgG and IgA in SARS CoV-2+ *vs* SARS-CoV-2− BAL. **(A)** S1-, S2-, RBD-, and NP-specific IgG and IgA responses in SARS-CoV-2+ BAL **(B)** S1-, S2-, RBD-, and NP-specific IgG and IgA responses in SARS-CoV-2− BAL. **(A, B)** Proportion of specific IgG or IgA over total IgG or IgA measured by ELISA. Specific (OD450)/total IgA or G (μg/ml) are shown. *p*-Values were calculated by using Mann–Whitney test: *, *p* < 0.05; **, *p* < 0.01; ***, *p* < 0.005. **(C)** Correlations between specific S1-, RBD-, S2-, and NP-specific IgG antibodies in SARS-CoV-2+ BAL (red dots) and SARS-CoV-2− BAL (gray dots) and onset of symptom to sampling date (days). **(D)** Correlations between S1-, RBD-, S2-, and NP-specific IgA antibodies SARS-CoV-2+ BAL (red dots) and SARS-CoV-2− BAL (gray dots) and onset of symptom to sampling date (days). **(E)** Correlation between specific S1, RBD, S2, and NP IgA and IgG antibodies in SARS-CoV-2+ BAL individuals. **(F)** Correlation between S1-, RBD-, S2-, and NP-specific IgA and IgG in SARS-CoV-2− BAL individuals. All correlations were calculated using Spearman’s test. RBD, receptor-binding domain; NP, nucleocapsid protein.

We found that 27%, 33%, 47%, and 50% of SARS-CoV-2+ BAL had IgG to S1, S2, RBD, and NP, respectively, and 33%, 40%, 53%, and 57% had IgA specific to S1, S2, RBD, and NP, respectively (**Figure 2A**). The S1-specific IgA and RBD-specific IgA were slightly higher than IgG (no statistical differences due to low sample numbers). Conversely, IgA specific for S2 and NP was statistically significantly higher than IgG (32 ± 7.6 for S2-IgA *vs* 5.5 ± 2.3 for S2-IgG, *p* = 0.01; 62 ± 3.2 for NP-IgA *vs* 3.7 ± 1.4 for NP-IgG, *p* = 0.03).

Similarly, late in COVID-19 infection when virus replication was resolved, 38%, 59%, 50%, and 20% of SARS-CoV-2 neg BAL had IgG to S1, S2, RBD, and NP, respectively, and 65%, 65%, 55%, and 26% had IgA to S1 and S2, RBD, and NP, respectively (**Figure 2B**).

The same IgA to IgG ratio was observed in late SARS-CoV-2 neg BAL samples as shown in **Figure 2B**, with S1-specific IgA statistically significantly higher than IgG (112 ± 30 *vs* 19 ± 6.4 respectively, *p* = 0.02). S2-specific IgA was higher than IgG (69.5 ± 19 *vs* 26 ± 10 respectively, *p* = 0.02), whereas anti-NP of both isotypes was present in an equal proportion of patients (55% *vs* 50%, respectively). Finally, following the same pattern, N-specific IgA was higher compared to IgG (mean N-specific IgA and IgG: 44 ± 13 *vs* 15 ± 6.2 respectively, *p* = 0.007).

We next correlated the anti-S1, RBD, S2, NP IgG and IgA responses with the onset of symptoms to sampling to evaluate the antibody kinetic. As shown in **Figure 2C**, SARS-CoV-2-specific IgG was detected early during the infection in SARS-CoV-2+ BAL. S1 and NP-IgG persisted during the disease and after viral clearance from the lung, whereas S2-IgG slowly decreased in SARS-CoV-2 neg BAL. RBD-specific IgG declined once the virus was eliminated from the BAL (**Figure 2C**, gray dots). Kinetics of the specific IgA responses mirrored that of the IgG: specific antibodies appeared at the initial phase of infection (SARS-CoV-2+ BAL, **Figure 2D**, red dots) and persist over time (SARS-CoV-2− BAL, **Figure 2D**, gray dots).

The specific level of BAL IgA anti-S1, anti-RBD, anti-S2, and anti-NP correlated with that of IgG in both the early (**Figure 2E**) and late phases (**Figure 2F**) of COVID-19. In SARS-CoV-2+ BAL, corelation between IgG and IgA was higher for S1 (0.8, *p* = 0.001) followed by RBD-specific response (r = 0.5, *p* = 0.001) (**Figure 2E**). The strongest correlation in SARS-CoV-2 neg BAL patients was observed for anti-S2 and anti-NP IgA and IgG (r = 0.6, *p* = 0.0002) followed by S1 (r = 0.4, *p* = 0.005, **Figure 2F**). These results show that IgG and IgA simultaneously evolve during severe COVID-19 development, independently of viral replication.

In summary, severe COVID-19 patients are capable of mounting a virus-specific mucosal immune response, which persists after virus elimination with higher levels of IgA than IgG.

### Bronchoalveolar lavages from COVID-19-infected individuals contain IgA-SARS-CoV-2 immune complexes

Unexpectedly, although in agreement with other studies on serum samples, a small but significant fraction (12%) of the BAL tested in our study had no detectable IgA against all antigens tested. In line with our findings, a recent study demonstrated that a high proportion of patients had neither detectable viral-specific IgG nor IgA in their nasopharyngeal compartments (37), although the reasons underlying these findings were not studied.

**Figure 3:**
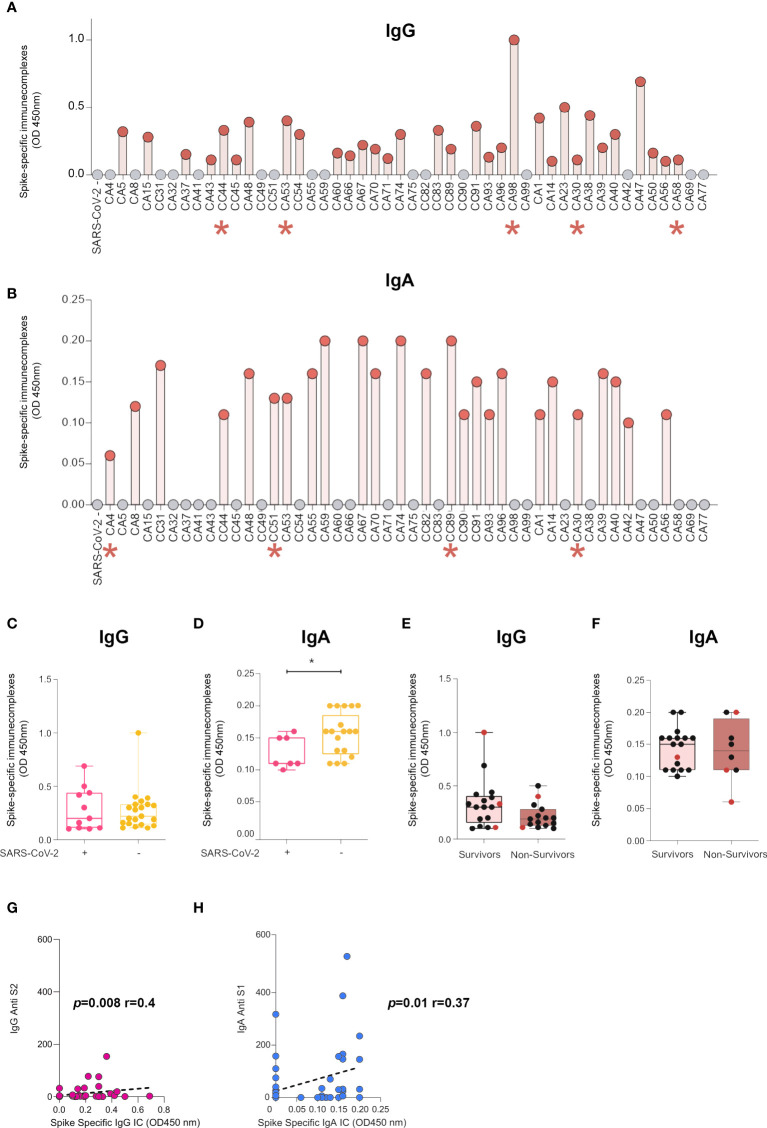
BAL from SARS-CoV-2-infected individuals contain IgG- and IgA-spike immune complexes (ICs). **(A)** Anti-spike IgG ICs were detected in 32 of the 48 samples analyzed by ELISA. Indicated with a red asterisk are individuals who had undetectable levels of IgG anti-spike/NP antibodies. **(B)** Anti-spike IgA ICs were detected in 25 of the 48 samples analyzed by ELISA. Indicated with red asterisk are individuals who had undetectable levels of IgA anti-spike/NP antibodies. **(C, D)** Comparison of presence of ICs made of spike with IgG (c) or IgA **(D)** in SARS-CoV-2+ BAL individuals and SARS-CoV-2− BAL subjects. *p*-Values were calculated by using Mann–Whitney test: *, *p* < 0.05. **(E, F)** Comparison of presence of ICs made of spike with IgG **(E)** or IgA **(F)** in survivors *vs* non-survivors. **(G, H)** Correlation between specific S1 and levels of ICs made of spike with IgG **(G)** or IgA **(H)**. Correlations were calculated using Spearman’s test. BAL, bronchoalveolar lavage; NP, nucleocapsid protein.

We hypothesized that the presence of IgG or IgA immune complexes (ICs) could prevent the detection of anti-SARS-CoV-2 antibodies in these samples, in line with the recent report that ICs are potential determinants of the cytokine storm in severe COVID-19 (38). We thus established an ELISA to measure IgG and IgA complexed with spike antigens (**Figure 3**).

IgG-spike ICs were detected in 32 of the 48 samples analyzed (66%, **Figure 3A**). Interestingly, five out of 11 individuals negative for specific IgG against S1, RBD, S2, and NP (CA4, CC31, CA32, CC44, CA53, CA59, CA98, and CA30) had detectable levels of IgG-spike ICs (CC44, CA53, CA98, CA30, and CA58; **Figure 3A**, red asterisks), indicating that spike-specific IgG is present in these BAL but remains associated with the virus or free spike. Regarding IgA, spike ICs were present in 25 of the 48 samples analyzed (52%, **Figure 3B**). Remarkably, four out of six individuals negative for specific IgA against S1, RBD, S2, and NP (CA4, CC51, CC89, CA30, CA58, and CA77) had detectable levels of IgA-spike ICs (CA4, CC51, CC89, and CA30; **Figure 3A**, red asterisks), mirroring what we measured in the case of IgG. IgG-spike ICs were detected in the same proportions in either the early or late phase of the infection (**Figure 3C**). On the contrary, IgA-spike ICs were predominant in individuals with no virus in BAL, in the late phase of infection (mean SARS-CoV-2+ BAL *vs* SARS-CoV-2 neg BAL 0.12 ± 0.009 *vs* 0.15 ± 0.007, *p* = 0.03, **Figure 3D**).

The presence of both IgG and IgA ICs was not associated with survival (**Figures 3E, F** respectively). However, we revealed that three out of the four individuals with detectable levels of IgA-spike ICs but undetectable anti-spike IgA underwent a fatal issue (**Figure 3F**, red dots).

Finally, anti-S2 IgG statistically correlated with IgG ICs (*p* = 0.008, r = 0.4, Spearman’s correlation, **Figure 3G**) and anti-S1 IgA with IgA ICs (*p* = 0.008, r = 0.4, Spearman’s correlation, **Figure 3H**).

Altogether, these results demonstrate that BAL from SARS-CoV-2-infected individuals contain IgA-spike ICs, which were more predominant in the late phase of the infection. Furthermore, IgA ICs might impair direct detection of spike-specific IgA by direct ELISA, and more importantly, IgA ICs in BAL might be adverse for patient disease development, most likely by stimulating myeloid cells *via* Fc-alpha receptors, as shown recently for IgG *via* Fc-gamma receptors (39, 40).

### Mucosal IgG and IgA targeting the receptor-binding domain from the ancestral SARS-CoV-2 cross-reacted with receptor-binding domain from emerging variants

The emergence of SARS-CoV-2 variants roused the question of whether the humoral response developed against the ancestral virus could offer cross-protection against the genetic variants. The spike protein is the main viral protein subjected to mutations, especially in the RBD, the principal spike subunit targeted by neutralizing antibodies. The N501Y mutation is the main mutation detected in the Alpha (B.1.1.7) variant that appeared in the United Kingdom (7, 41). The B.1.1.7 variant emerged independently in South Africa (42), whereas the Gamma variant (P.1) appeared in Brazil due to travelers from Japan and the B.1.617.2 Delta variant in India (43). A significant cross-protection of vaccinated individuals is detected against these new variants (44–46). The cross-reactivity of mucosal antibodies elicited from patients during natural SARS-CoV-2 infection remains unknown.

**Figure 4:**
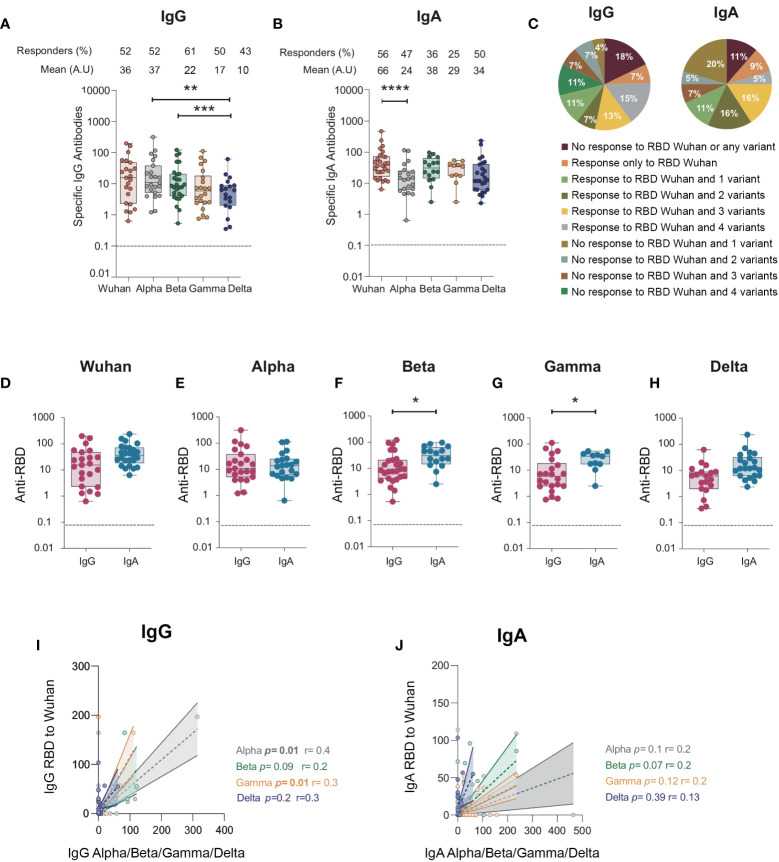
IgG and IgA antibodies from BAL from SARS-CoV-2-infected individuals against RBD protein from SARS-CoV-2 Alpha (B.1.1.7), Beta (B.1.351), Gamma (P.1), and Delta (B.1.617) variants. **(A)** Specific IgG responses against Alpha, Beta, Gamma, and Delta RBD in BAL from SARS-CoV-2-infected individuals. **(B)** Specific IgA responses against Alpha, Beta, Gamma, and Delta RBD in BAL from SARS-CoV-2-infected individuals. **(A, B)** Proportion of specific IgG or IgA over total IgG or IgA measured by ELISA (specific (OD450)/total IgA or G (μg/ml)) are shown. **(C)** Pie charts showing the percentages of the different responses of IgG (left) and IgA (right) to Wuhan RBD and the different variants. **(D–H)** Comparison between specific IgG and IgA responses detected against the RBD from ancestral Wuhan strain **(D)**, Alpha **(E)**, Beta **(F)**, and Gamma **(G)**and Delta **(H)** variants. Correlation between IgG **(I)** and IgA **(J)** specific to Wuhan RBD and IgG and IgA antibodies specific for Alpha, Beta and Gamma variants. Correlations were calculated using Spearman’s test. *p*-Values were calculated by using Wilcoxon test. *, *p* < 0.05; **, *p* < 0.01; ***, *p* < 0.005, ****, p < 0.0001. Dashed line: cutoff value for antibody detection. BAL, bronchoalveolar lavage; RBD, receptor-binding domain.

Therefore, we evaluated whether mucosal anti-RBD IgA and IgG elicited toward the Wuhan virus in the BAL we collected would cross-react with the RBD from the Alpha (B.1.1.7), Beta (B.1.351), Gamma (P.1), and Delta (B.1.617) variants. When tested in ELISA, >50% of the BAL samples had IgG cross-reacting with the Alpha, Beta, and Gamma RBD variants (52% to Alpha, 61% to Beta, and 50% to Gamma; **Figure 4A**), whereas 43% cross-reacted with the Delta RBD variant, all compared with 52% of the Wuhan ancestral RBD (**Figure 4A**). Moreover, quantitatively, the level of IgG specific to the Delta RBD was lower than that of the Alpha (mean, 10 ± 3.1 *vs* 37 ± 14 respectively, *p* = 0.005) and the Beta ones (mean, 10 ± 3.1 *vs* 22 ± 5.9 respectively, *p* = 0.0005). In contrast, IgA targeting the Alpha, Beta, and Gamma RBD variants was only detected in 25% to 47% of the study population (**Figure 4B**). However, 50% of the samples had IgA specific to Delta RBD (**Figure 4B**), compared with the 43% IgG response. Of note, 18% and 11% of the individuals failed to raise IgG and IgA, respectively, against any of the variants or to the RBD Wuhan variant. In contrast, 15% and 5% of the individuals had IgG and IgA against all variants (including the ancestral Wuhan one), respectively. Finally, only 7% and 9% of the individuals developed IgG and IgA, respectively, to the sole Wuhan RBD (**Figure 4C**).

The magnitude of the IgA response toward RBD was higher than that of the IgG for the Beta, Gamma, and Delta variants (mean for anti-Beta RBD response: 38 ± 7 *vs* 21 ± 6, respectively, *p* = 0.02; mean for anti-Gamma RBD response: 29 ± 5 *vs* 17 ± 6 respectively, *p* = 0.0; mean for anti-Delta RBD response: 8 ± 3 *vs* 28 ± 10 respectively, *p* = 0.01, **Figures 4F–H**), as observed for the Wuhan strain (**Figure 4D**). In contrast, no differences were observed for the Alpha variant (**Figure 4E**). Finally, when analyzed at the individual sample level, only the magnitude of IgA to RBD Alpha (*p* = 0.01, r = 0.34, Spearman’s correlation, **Figure 4I**) and Gamma (*p* = 0.01, r = 0.3, Spearman’s correlation, **Figure 4J**) correlated.

Altogether, our results showed that i) in the majority of BAL, IgG and IgA had cross-variant neutralization capacity; ii) Beta, Gamma, and Delta RBD-specific IgA are higher than IgG in line with what was observed for Wuhan RBD-specific antibodies; iii) 11% to 18% of the individuals developed neither IgG nor IgA to any variant RBD studied; iv) conversely, 5% to 15% of the individuals developed IgG and IgA to all RBDs studied.

### Bronchoalveolar lavages neutralized SARS-CoV-2 infection more efficiently earlier than at the later stage of disease *in vitro*

**Figure 5:**
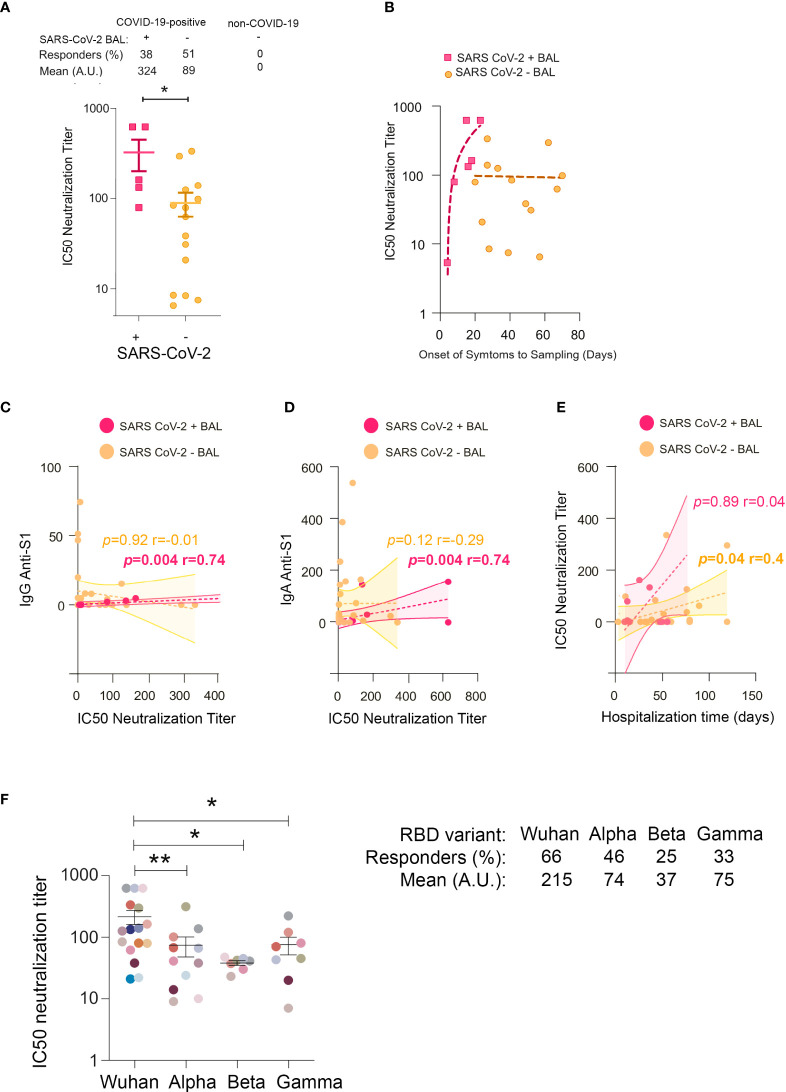
IC50 neutralization titers in BAL from COVID-19 individuals. **(A)** Comparison of IC50 neutralization titers between SARS-CoV-2+ BAL and SARS-CoV-2− BAL samples. **(B)** Correlations between IC50 neutralization titers and the onset of symptoms to sampling date in SARS-CoV-2+ BAL (light blue squares) and SARS-CoV-2− BAL (purple dots) individuals. **(C)** Correlation between IC50 neutralization titers and spike-specific IgG in SARS-CoV-2+ and SARS-CoV-2− BAL. **(D)** Correlation between IC50 neutralization titers and spike-specific IgA in SARS-CoV-2+ and SARS-CoV-2− BAL. **(E)** Correlation between neutralization activity and hospitalization time in SARS-CoV-2+ and SARS-CoV-2− BAL. **(F)** IC50 neutralization titers of BAL from SARS-CoV-2+ individuals against ancestral Wuhan, and Alpha, Beta, and Gamma SARS-CoV-2 variants. A specific color is associated with each individual. All correlations were calculated using Spearman’s test. *p*-Values were calculated by using Mann–Whitney test. *, *p* < 0.05; **, *p* < 0.01. BAL, bronchoalveolar lavage.

To gain insight into the functions of mucosal SARS-CoV-2-specific IgA and IgG, we evaluated BAL neutralization activities. First, we compared the IC50 neutralization titers of SARS-CoV-2+, SARS-CoV-2 neg, and non-COVID-19 BAL. We found that 38% and 51% of virus containing and virus lacking BAL neutralized SARS-CoV-2 infection, respectively, whereas BAL from non-COVID-19 patients lacked neutralizing activity (**Figure 5A**). IC50 neutralization titers were statistically significantly higher in SARS-CoV-2+ compared with SARS-CoV-2 neg BAL (mean 324 ± 123 *vs* 89 ± 26 respectively, *p* = 0.01). This indicated that neutralizing antibodies developed early after infection when the virus replicates and decreased later after the virus has been cleared from the lung. Accordingly, the IC50 neutralization titers, plotted as a function of onset of symptoms to sampling time, first sharply rose during the first 3 weeks of the disease before slowly declining (**Figure 5B**). For ethical reasons, we could not collect enough samples from severe COVID-19 patients to purify each antibody isotype from these mucosal fluids. To attribute the neutralization observed to one isotype, we had to rely on correlations. Early in infection, IC50 neutralization titers of SARS-CoV-2+ BAL positively correlated with S1-specific IgG and IgA (**Figures 5C, D** respectively).

The presence of neutralizing activity has been associated with a worse outcome in many studies (47–49). To address this issue, hospitalization duration for each individual was calculated (**Supplementary Figure 2A**) and correlated with the corresponding BAL neutralization titers. A positive correlation was only observed between IC50 and patient hospitalization duration for SARS-CoV-2 neg BAL (*p* = 0.04, r = 0.4, **Figure 5E**), suggesting that long-lasting mucosal neutralizing antibodies may be disadvantageous for patient recovery. Accordingly, the hospitalization duration was shorter for individuals with SARS-CoV-2+ compared to SARS-CoV-2 neg BAL (mean duration: 35 ± 5.8 *vs* 49 ± 5.4 days). Indeed, the former showed sharp neutralizing antibodies rise cross-sectionally, whereas the latter had a stabilized neutralizing response.

Altogether, the early mucosal neutralizing response might exert a protective function; conversely, in the later phases associated with virus clearance from the pulmonary mucosa, additional non-neutralizing roles of neutralizing antibodies might be responsible for adverse effects (50). More analyses in larger cohorts of patients are required to confirm this conclusion.

### Broncho alveolar lavages from severe COVID-19 patients infected by the ancestral SARS-CoV-2 neutralize later SARS-CoV-2 variants

Neutralization activities against RBD from the Wuhan (WT), Alpha (B.1.1.7), Beta (B.1.351), and Gamma (P.1) variants were evaluated in the BAL from 14 SARS-CoV-2+ and 10 SARS-CoV-2 neg patients; neutralization activities against all the viruses considered were detected in 66%, 46%, 25%, and 33% of the individuals, respectively (**Figure 5F**).

As expected, neutralization titers against the Wuhan virus were higher compared to those against the Alpha (mean 215 ± 55 and 74 ± 27 respectively, *p* = 0.004), Beta (mean 215 ± 55 and 37 ± 3.2 respectively, *p* = 0.01), and Gamma (mean 215 ± 55 and 75 ± 24 respectively, *p* = 0.02) variants (**Figure 5F**).

Altogether, these data supported our previous findings showing that the majority of BAL contained mucosal antibodies to viral variants indicative of a potent cross-neutralization capacity.

### Non-survivors developed persistent SARS-CoV-2 spike and NP-specific IgG and S1-specific IgA

**Figure 6:**
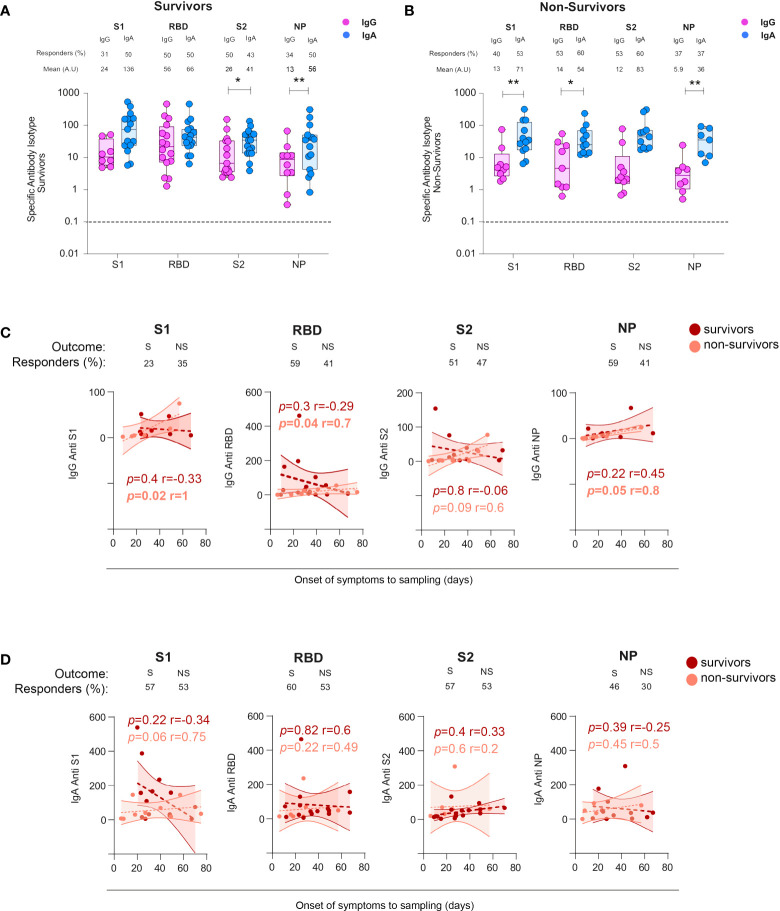
Specific IgG and IgA responses in COVID-19+ survivors *vs* non-survivors. **(A)** Specific S1-, S2-, RBD-, and NP-specific IgG and IgA responses in survivors and non-survivors. *p*-Values were calculated by using Wilcoxon test **(A, B)** Dashed line: cutoff value for antibody detection. **(C)** Comparison of the kinetics from the cross-sectional SARS-CoV-2-specific IgG responses in survivors (S) *versus* non-survivors (NS). **(D)** Comparison of the kinetics from the cross-sectional SARS-CoV-2-specific IgA responses in survivors (S) *versus* non-survivors (NS). All correlations were calculated using Spearman’s test. *, *p* < 0.05; **, *p* < 0.01. RBD, receptor-binding domain; NP, nucleocapsid protein.

To investigate whether mucosal SARS-CoV-2-specific antibodies could play a role in the patient’s survival, we stratified BAL samples according to the outcome of patients, referred to as survivors and non-survivors. In survivors, the S2- but not S1- or RBD-specific IgA response was statistically higher compared with corresponding IgG (mean 41 ± 7.9 and 26 ± 10.5, respectively, *p* = 0.02, **Figure 6A**). Conversely, in non-survivors (**Figure 6B**), S1- and RBD-specific but not S2-specific IgA predominated over IgG (mean S1-IgA: 71 ± 26 *vs* S1-IgG: 13 ± 7.8, *p* = 0.007; mean S2-IgA: 83 ± 30 *vs* S2-IgG: 12 ± 7.7, *p* = 0.009). The dominant NP-specific IgA responses occurred independently of survival (in survivors: mean anti-NP-IgA: 56 ± 21 *vs* anti-NP IgG: 13 ± 3.2, *p* = 0.007, **Figure 6A**; in non-survivors, mean anti-NP-IgA: 36 ± 12, *vs* anti-NP IgG 5.9 ± 2.7 *p* = 0.01, **Figure 6B**).

However, analyses of the IgA or IgG S-specific responses varied with the patient outcome with S2-specific IgG being statistically significantly higher in survivors compared with non-survivors (mean S2-IgG equal to 26 ± 4.0 *vs* 12 ± 7.7, in survivors *vs* non-survivors, *p* = 0.03, **Supplementary Figure 3A**). Conversely, the S2-specific IgA response was statistically significantly higher in non-survivors compared with survivors [mean S2-IgA survivors, 41 ± 10 *vs* IgG anti-S2 non-survivors: 83 ± 30, *p* = 0.01 *(* **Supplementary Figure 3B**)].

In a cross-sectional analysis, we then investigated whether the kinetics of specific antibodies might vary with patient survival. We found that the levels of IgG and IgA toward S1, RBD, S2, and NP were similar, irrespective of survival (mean of 33 ± 3.3 *vs* 33 ± 4.5 days for survivors and non-survivors, respectively, **Supplementary Figure 2B**). In non-survivors only, S1-, RBD-, S2-, and NP-specific IgG and S1-specific IgA increased over time (**Figures 6C, D**), Moreover, surviving individuals had higher but transient S2 specific IgG response in the lungs (**Figure 6C**). These results highlighted that antibody persistence, but not their amount, might play an adverse role in COVID-19 pathogenesis.

### In contrast to Survivors, Non-survivors lose neutralizing antibody response over time

**Figure 7:**
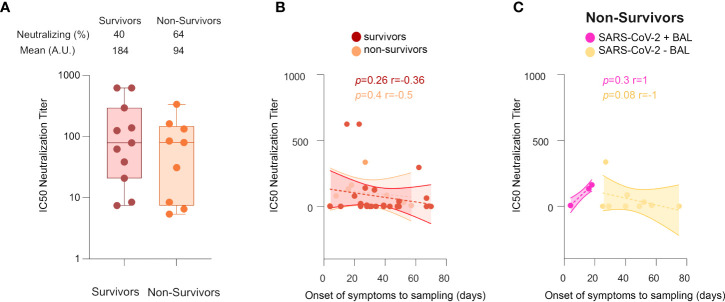
Neutralization activities in COVID-19+ survivors *vs* non-survivors. **(A)** Comparison between IC50 neutralization titers between survivors and non-survivors. **(B)** Cross-sectional representation of neutralizing antibodies in survivors *vs* non-survivors, shown as correlation between IC50 neutralization titters and time from symptom onset to sampling date in survivors and non-survivors. **(C)** Cross-sectional representation of neutralizing antibodies in non-survivors, shown as correlation between IC50 neutralization titters and time from symptom onset to sampling date, in SARS-CoV-2+ BAL and SARS-CoV-2− BAL individuals. All correlations were calculated using Spearman’s test. *p*-Values were calculated by using Wilcoxon test. BAL: bronchoalveolar lavage.

We next evaluated the involvement of neutralizing antibodies in patient outcomes. For individuals developing neutralizing activities in BAL, the neutralization titers in survivors and non-survivors were not statistically significantly different (mean equal to 184 ± 70 *vs* 94 ± 35, respectively, **Figure 7A**); and no differences in the kinetics of the neutralizing response between survivors and non-survivors were detected (**Figure 7B**). However, in non-survivors (**Figure 7C**), the IC50 neutralizing titers started to rise in direct correlation with time (from the onset of the disease to sampling) in SARS-CoV-2+ BAL (r = 1, *p* = 0.3), whereas it decreased in SARS-CoV-2− BAL (r = −1, *p* = 0.08). Of note, in both cases, the correlation was not statistically significant due to the small sample size. These data suggest that, in severe COVID-19 patients, a strong neutralizing activity mounted at the early phase of the disease when the virus replicates, followed by its decrease when the virus disappears is not sufficient. As previously speculated, mucosal antibodies might have additional activities, likely contributing to the fatal outcome of these patients (51, 52), although more samples need to be analyzed to confirm this hypothesis. In this context, some BAL samples were tested for antibody-dependent enhancement (ADE) activity using the protocol established by Wu et al. (53), but no ADE activity was detected (data not shown).

### B cell subsets remained stable over the course of the disease

Mucosal antibodies are raised locally after mucosa-specific homing of B cells (54). Therefore, we characterized B-cell populations in BAL from our cohort. Five B-cell populations were analyzed by flow cytometry: i) CD27- CD21+ naïve B, ii) CD27+ CD21- activated memory B cells, iii) CD38+ CD138+ plasma B cells, iv) CD27+ CD21+ resting memory B cells, and vi) CD27- CD21+ tissue memory B cells. In addition, B cells were stratified according to the presence of IgG and IgA (as shown in the gating strategy, **Supplementary Figure 1**).

**Figure 8:**
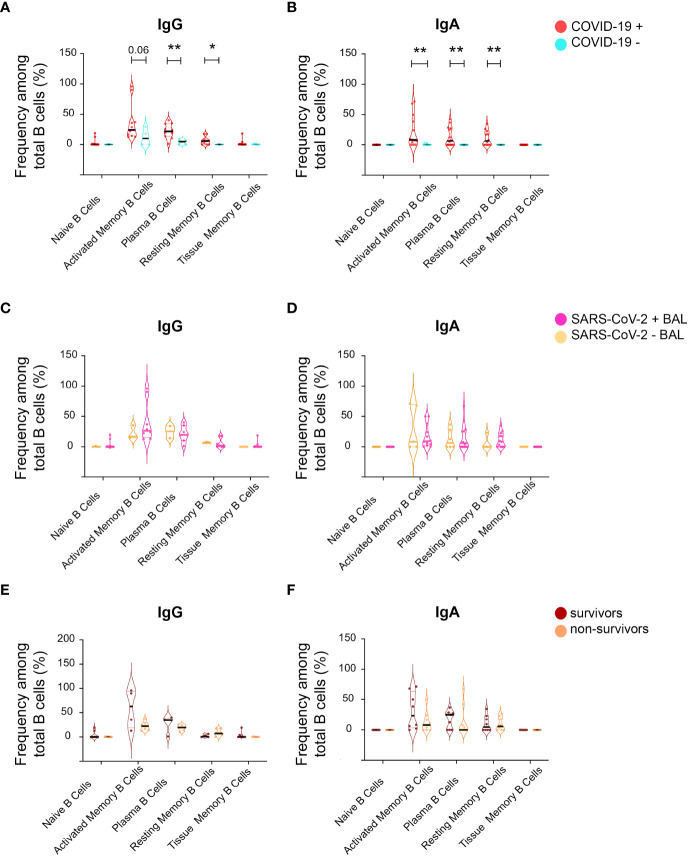
Analysis of B-cell phenotype in BAL supernatant from SARS-CoV-2-infected individuals. From total B cells, five B-cell populations were defined including Naïve B cells, activated memory B cells, plasma B cells, resting memory B cells, and tissue memory B cells according to a gating strategy shown in **Supplementary Figure 1**, which were further labeled for IgG (IgG+) and IgA (IgA+). **(A)** Frequencies of different IgG+ B-cell populations between COVID-19+ and COVID-19− individuals in the total B-cell population shown as violin plots. **(B)** Frequencies of different IgA+ B-cell populations between COVID-19+ and COVID-19− individuals in the total IgA+ B-cell population shown as violin plots. **(C)** Frequencies of different IgG+ B-cell populations between SARS CoV-2+ BAL and SARS CoV-2− BAL individuals in the total IgG+ B-cell population shown as violin plots. **(D)** Frequencies of different IgA+ B-cell populations between SARS CoV-2+ BAL and SARS CoV-2− BAL individuals in the total IgA+ B-cell population shown as violin plots. **(E)** Frequencies of different IgG+ B-cell populations between survivors and non-survivors in the total IgG+ B-cell population shown as violin plots. **(F)** Comparison of different IgA+ B-cell populations between survivors and non-survivors in the total IgA+ B-cell population shown as violin plots. p-Values were calculated by using Mann–Whitney test *, p < 0.05; **, p < 0.01.

IgG plasma B-cell proportion was higher in BAL from COVID-19 compared to non-COVID-19 subjects (mean 22 ± 33.8 *vs* 3.9 ± 1.7 *p* = 0.004, **Figure 8A**). A trend toward a higher proportion of activated and resting memory B cells in infected compared with non-infected was also observed (**Figure 8A**). Similarly, IgA activated, plasma, and resting memory B-cell proportions were higher in COVID-19 than in non-COVID-19 subjects (**Figure 8B**). The percentages of IgA and IgG naïve, activated, plasma, resting, and tissue memory B-cell proportions remained similar irrespective of virus detection in BAL (**Figures 8C, D**) and did not have an impact on COVID-19 outcome (**Figures 8E, F**).

### Cytokine levels in bronchoalveolar lavages from SARS-CoV-2-infected individuals

**Figure 9:**
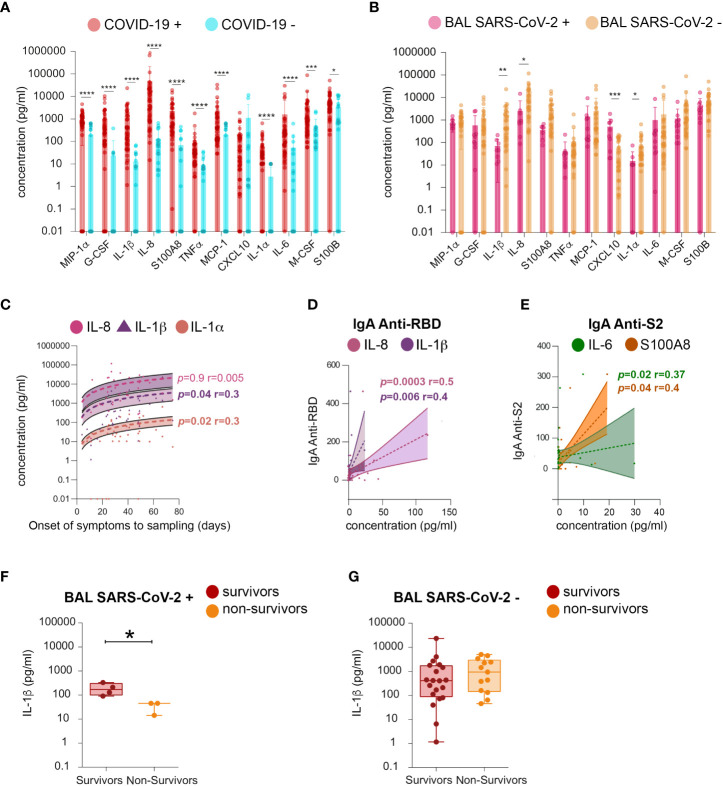
Analysis of cytokines in BAL fluid. Quantification of the following cytokines in BAL from SARS-CoV-2-infected individuals: MIP-1α, G-CSF, IL-1β (IL-1β), IL-8, S100A8, TNF-a, MCP-1, CXCL10, IL-1α (IL-1A), IL-6, M-CSF, and S100B. **(A)** Mean amounts of cytokines (pg/ml) evaluated between COVID-19+ and COVID-19− individuals. **(B)** Mean amounts of cytokines (pg/ml) evaluated between BAL SARS CoV-2+ and BAL SARS CoV-2− individuals. **(C)** Cross-sectional concentration (pg/ml) of IL-8, IL-1β, and IL-1A as function of time from onset of symptoms to sampling date. **(D)** Correlation between RBD-specific IgA (shown as proportion of specific IgG or IgA over total IgG or IgA measured by ELISA (specific (OD450)/total IgA or G (μg/ml)) and IL-1β and IL-8 concentration (pg/ml). **(E)** Correlation between S2-specific IgA (shown as proportion of specific IgG or IgA over total IgG or IgA measured by ELISA (specific (OD450)/total IgA or G (μg/ml)) and S100A8 and IL-6 concentration. **(F)** Comparison of the levels of IL-1β (pg/ml) between survivors and non-survivors in SARS-CoV-2+ BAL individuals. **(G)** Comparison of the levels of IL-1β (pg/ml) between survivors and non-survivors in individuals BAL SARS-CoV-2−. *p*-Values were calculated by using Mann–Whitney test. *, *p* < 0.05, **, *p* < 0.01; ***, *p* < 0.005, ****, *p* < 0.0001. BAL, bronchoalveolar lavage; G-CSF, granulocyte colony-stimulating factor; RBD, receptor-binding domain.

The following cytokines related to inflammation and B-cell response were quantified: MIP-1 alpha, G-CSF, IL-1β, IL-8, S100A8, TNF-alpha, MCP-1, CXCL10, IL-1α, IL-6, M-CSF, and S100B. BAL from SARS-CoV-2-infected individuals had higher levels of MIP-1 alpha, G-CSF, IL-1β, IL-8, S100A8, TNF-alpha, MCP-1, IL-1α, IL-6, M-CSF, and S100B, compared to those of uninfected ones (**Figure 9A**). The levels of all cytokines persisted after virus elimination from BAL (**Figure 9B**). Moreover, IL-1α, IL-1β, and IL-8 were statistically significantly higher in virus-free compared to virus-containing BAL, and their levels increased during disease progression (mean IL-1α SARS-CoV-2+ BAL *vs* SARS-CoV-2 neg BAL 15 ±7.7 *vs* 72 ± 21 respectively, *p* = 0.02; mean IL-1β: 1,815 ± 718 *vs* 71 ± 24, respectively, *p* = 0.01; mean IL-8: 14,255 ± 4,167 *vs* 2,540 ± 1,543 respectively, *p* = 0.04). These data indicated that the persistence of these cytokines is independent of the presence of the virus in the BAL. Conversely, an opposite scenario was observed for CXCL-10 (**Figure 9A**): high levels of CXCL-10 were detected in SARS-CoV-2+ BAL and then decreased with time (mean 591 ± 187 *vs* 52 ± 12, respectively, *p* = 0.001). The kinetics of the cytokine level revealed a positive correlation between the amounts of IL-1β and IL-1α and the time from symptom onset to sampling (IL-1β: *p* = 0.04, r = 0.3; IL-1α: *p* = 0.02, r = 0.3, **Figure 9C**). Additionally, both IL-1β and IL-8 correlated with RBD-specific IgA (IL-1β *p* = 0.006 r = 0.4, IL-8 *p* = 0.0003 r = 0.5, **Figure 9D**), whereas both S100A8 and IL-6 correlated with S2-specific IgA (S100A8 *p* = 0.04 r = 0.4, IL-6 *p* = 0.02 r = 0.37; **Figure 9E**). Higher levels of IL-1β were found in BAL from survivors but only when the virus replicates (mean survivors *vs* non-survivors 102 ± 40 *vs* 15 ± 7.7, *p* = 0.05, **Figures 9F, G**). Therefore, patients having low levels of IL-1β in SARS-CoV-2+ BAL might be prone to develop fatal COVID-19 as compared to those with high levels. In contrast, at a later phase of the disease, IL-1β levels might not affect survival.

These results were confirmed by applying Bayesian logistic regression on BAL cytokines measurements to model the association of the odds ratio of non-surviving against surviving probability (referred to as odds ratio). Cytokines were significantly associated with the odds ratio only when samples were further stratified according to the phase of the disease. In agreement, only IL-1β was statistically significantly associated with the odds ratio early in infection. Indeed, the estimated one-sided 97.5% credible interval of its coefficient was less than 1, indicating it was a negative risk factor. IL-1β loses its association with the odds ratio later in the disease. These data suggest that a higher level of lung IL-1β might be predictive of patient survival only during the early phase of infection. Additional studies using longitudinal sampling are needed to definitively establish whether IL-1β could be a marker of severity during the course of the diseases.

## Discussion

The humoral immune response against SARS-CoV-2 has been extensively evaluated in the serum of COVID-19 individuals (8), but very limited data exist on the mucosal immune response, including that in the respiratory tract, the main portal of entry and the replication site of the virus. Previous studies on SARS-CoV-2 humoral immunity have been conducted addressing compartments other than BAL, namely, saliva (55, 56) and tears (57), although these fluids are not produced at the primary site of infection. Conversely, BAL fluid is representative of the pulmonary microenvironment in terms of lung cell types, lung cytokines, and mucosal antibodies.

In this study, we profiled the mucosal humoral immune response to SARS-CoV-2 spike and NP in BAL from severe COVID-19 patients and evaluated their neutralizing activity. BAL were stratified according to the presence or absence of SARS-CoV-2, corresponding to an early phase of the disease lasting 3 weeks [longer than reported for mild COVID-19 (58)] and a later phase lasting more than five additional weeks. We found that sustained levels of non-neutralizing S1, RBD, S2, and NP-IgG and S1-IgA were associated with fatal outcomes once SARS-CoV-2 was cleared from the lungs. Conversely, at the early stages of viral replication, high levels of IL-1β in the BAL might be associated with survival.

Previous studies on sera from COVID-19 patients have shown that IgA is prevalent in the early SARS-CoV-2 systemic humoral responses (13). These circulating monomeric IgA antibodies appeared from day 4 after the onset of symptoms, peaked at week 3, and persisted longer than IgM (12). Importantly, Wang et al. (59) showed that plasma RBD-specific IgA had lower neutralizing activities than their IgG counterpart. Conversely, dimeric IgA engineered from monomeric RBD-specific IgA neutralized on average 15 times more SARS-CoV-2 than the monomeric form, suggesting the importance to study the native secretory form of IgA in mucosal lung fluids. Furthermore, in an integrated analysis of SARS-CoV-2 spike-specific antibodies, cytokines, viral load, and bacterial communities in paired nasopharyngeal swabs and plasma samples from a cohort of clinically distinct COVID-19 patients during acute infection, differential compartmentalization of the SARS-CoV-2 immune responses was reported (37).

The mucosal humoral responses in BAL contain secretory IgA and IgG, produced locally in the mucosa prior to secretion in the alveolar space, and have an antigenic repertoire distinct from the serum humoral response (14, 26, 60). We found that both SARS-CoV-2-specific IgA and IgG responses developed simultaneously after a week of infection when the virus replicates in BAL. However, when the virus is cleared from the lung mucosa at later stages of COVID-19, virus-specific IgA predominates over IgG. In agreement, the mucosal virus-specific IgA response has been detected early after infection, at day 6 post symptoms onset (40). Such lung IgA response was higher than that detected in serum samples (13) most likely because SARS-CoV-2 infection initiated in the nasal mucosa propagates rapidly to the lung initiating a local immune response. Conversely, the mucosal spike- and NP-specific IgG responses developed later, from day 18 post-symptom onset, whereas in the serum, RBD-specific IgG emerged at day 11, peaking at day 23 (61, 62). In our cross-sectional analysis of BAL from severe COVID-19 patients, all specific IgA responses increased from the initial phase of the infection. After the virus has been cleared from the lung S1-specific IgA notably decreased, whereas RBD-, S2-, and NP-specific IgA persisted or slowly decreased, in agreement with a sustained detection of IgA- and IgG-specific B-cell populations.

Our findings revealed that IgA might also play an adverse role in SARS-CoV-2 infection, as we have recently reported for serum IgA in a different cohort of patients (18). Indeed, we found that non-survivors developed higher amounts of S1- and RBD-specific IgA than IgG as compared to survivors, and S1-IgA increased over time in these subjects. However, BAL S1-, RBD-, S2-, and NP-specific IgG developed over time in non-survivors. Thus, virus-specific IgG might contribute to a worthy outcome by exacerbating mucosal innate immune cells such as alveolar macrophages *via* their respective Fc receptors (39, 40), an interaction reinforced by a lack of antibody fucosylation as observed for serum antibodies (39, 63, 64).

Together with IgG that stimulates the innate immune inflammatory response by interacting with Fc-gamma receptors, IgA contributed to this inflammatory pathway. Upon opsonization of bacteria, IgA binds to its receptor FcαRI (CD89), resulting in a cross-talk with Toll-like receptors (TLRs) that, in turn, lead to the production of pro-inflammatory cytokines (TNF-alpha, IL-1β, IL-6, and IL-23) by human macrophages, monocytes, and Kupffer cells (65). FcαRI is expressed by monocytes and several macrophage subsets including alveolar ones. Accordingly, we might speculate that virus-specific mucosal IgA forms immune complexes with the spike. These complexes might bind to FcαRI on macrophages, triggering a persistent cytokine storm, as suggested for IgG (39). Accordingly, IgA was abundant in a fraction of BAL from severe COVID-19 patients that we analyzed. However, additional studies are necessary to confirm this hypothesis.

A robust, although delayed, level of serum IgA, although IgG-independent, has been previously associated with a worse outcome and disease severity (13, 65, 66). This evidence reinforces the importance of antibody compartmentalization, which role might differ between serum and mucosa, likely due to antibody isotype fine antigenic specificities as reported recently in COVID-19 (37).

S2-specific mucosal IgA levels correlated positively with inflammatory cytokines present in BAL such as S100A8 and IL-6. Accordingly, pulmonary IgA developing at the primary site of SARS-CoV-2 infection may participate in virus-driven hyperinflammation, a phenomenon that is strongly correlated with COVID-19 mortality. In particular, increased levels of IL-6 observed in individuals with fatal COVID-19 (67) might favor isotype switching of mucosal B cells to IgA. Additionally, soluble IgA could have induced IL-6 production by normal human lung fibroblasts, together with other cytokines (IL-8, MCP-1, and GM-CSF) (68). This bidirectional interaction may create an autocrine loop, thereby participating in the uncontrolled cytokine storm driving fatal outcomes in COVID-19 patients. Moreover, pulmonary S1-specific IgA strongly positively correlated with IL-8 levels, which could contribute to the hyperinflammation and increase the mucosal antigen-specific antibodies (69), providing a potential biomarker of COVID-19 severity.

The emergence of SARS-CoV-2 variants, including those in the United Kingdom (Alpha, B.1.1.7), South Africa, (Beta, B.1.351), Brazil (Gamma, P.1), and India (Delta, B.1.617) induced serious concerns worldwide about the capability of SARS-CoV-2 antibodies raised by natural infection or vaccination to offer cross-protection.

Previous infection with other coronaviruses could play a role in the development of cross-reacting antibodies, although only 1% of these individuals developed RBD-specific antibodies more commonly observed for the SARS-CoV-2 full-length S and against the NP protein (70). Preexisting, cross-reactive antibodies preferentially target specific, immunodominant epitopes located in functional sites of the S2 subunit (71). Finally, antibodies against other human coronaviruses (HCoV) are also boosted by SARS-CoV-2 infection, particularly during severe COVID-19 illness (72). Whether these cross-reactive antibodies confer any protection against infection or whether they modulate disease severity is unclear. One report found that levels of pre-pandemic or pre-infection cross-reactive SARS-CoV-2-binding antibodies did not correlate with protection from SARS-CoV-2 infection and hospitalization (70), while others found opposite results (73). Altogether, these studies highlighted that factors other than serum antibodies might play a role in cross-protection, including T-cell responses and cross-protective mucosal antibody responses.

Fifty percent of BAL samples contained IgG cross-reacting with the Alpha, Beta, and Gamma RBD variants, whereas 43% cross-reacted with the Delta RBD variant, all compared with 51% of the Wuhan RBD. In contrast, IgA specific to the Alpha, Beta, and Gamma RBD variants was detected in BAL from 25% to 47% of the studied population. These data are in line with the detection of RBD-specific IgA and IgG in BAL from five and four out of eight patients as reported by Sterlin et al. (12, 13).

Surprisingly, 50% of our BAL samples collected when the Wuhan virus was circulating had IgA specific to Delta RBD, suggesting that infection with the Wuhan virus induced a strong cross-reactivity response. This may have contributed to the lack of reinfection cases in countries when the Delta variant predominated. Mucosal antibodies induced by Wuhan virus infection were also largely cross-reactive for other variants, as the few individuals we studied developed lung IgG or IgA targeting only the RBD from the ancestral lineage (7% and 9%, respectively), whereas 15% and 5% had IgG and IgA, respectively, against all variants.

Altogether, this set of results suggested that a previous severe COVID-19 might confer cross-protection to re-infection with at least the Alpha, Beta, and Gamma variants at the site of viral entry, in agreement with the epidemiological data recorded and mentioned earlier (44, 45). Our data present an encouraging scenario in which individuals vaccinated with Wuhan spike-based vaccines may be protected from infection with SARS-CoV-2 variants.

At the functional level, neutralization titers are higher in individuals with SARS-CoV-2 in the BAL early in infection likely resulting from anti-S1 IgG and IgA activities, as indicated by their positive correlation. In agreement with the present data, neutralizing antibodies have been identified in serum from COVID-19 patients (3), and the BAL from patients with severe COVID-19 showed a similar IC50 neutralizing titer range (12, 13). Later in the disease, neutralizing antibodies strongly decrease, probably because the antibody-mediated antiviral activity is not required when the virus has been cleared.

The relationship between the presence of virus-specific neutralizing activity in the sera and the patient outcome remains controversial. Neutralizing titers have been reported in asymptomatic individuals (74, 75). A rapid decline in the neutralizing response (47) or a decline within 3 months following SARS-CoV-2 infection was observed (76) in larger longitudinal cohorts, and neutralizing titers strongly correlated with disease severity (47). We now report that mucosal antibody neutralizing activities are similar, independently of the patient outcome or hospitalization time. Accordingly, the function of SARS-CoV-2-specific reported antibodies in serum and mucosal compartments differs, as in COVID-19 (37) and other pathologies, such as HIV (77). Regarding the kinetics of the neutralizing antibodies, a rapid decay in serum anti-SARS-CoV-2 antibodies in patients has been reported (78, 79). Similarly, we found that at the mucosal lung level, neutralizing antibodies decreased over time but over a longer period compared with blood (78, 79). Furthermore, the levels of S1-, RBD-, S2-, and NP-specific mucosal IgG and S1-specific mucosal IgA in non-survivors with no virus in BAL persist, suggesting that persistent spike- and NP-specific antibodies are non-neutralizing. These antibodies might rather contribute *via* their interaction with FcαR on innate immune cells to this long-lasting severe COVID-19 state (39, 63–65).

At the pulmonary level, we found that SARS-CoV-2 infection increased the IgG plasma B cells as well as IgA activated, plasma, and resting memory B cells, although no differences were observed along with disease development or outcome. Accordingly, in a recent cross-sectional study of 188 recovered COVID-19 cases, the frequencies of SARS-CoV-2 spike-, RBD, and NP-specific memory B cells increased over the first 4 months post symptom onset. In agreement with our data, the development of circulating B-cell memory to SARS-CoV-2 was robust and likely long-lasting (19). Although we could not detect differences in whole B-cell phenotypes between survivors and non-survivors, differences might have been observed for SARS-CoV-2 antigen-specific B cells. Indeed, the analysis of S-specific B cells resulted in a more complex phenotype than previously expected. It combines two synchronous responses, each with individual dynamics during the extra-follicular reaction (19, 21), with mobilization of near-germline B-cell clones specific for SARS-CoV-2 S protein. In addition, these B cells could correspond to preexisting highly mutated memory B specific for the S protein of other seasonal beta-coronaviruses. Furthermore, we cannot exclude the presence of mucosal IgA-specific B-1 cells in BAL (80). Hence, in addition to secreting IgA (81), B-1 cells might have additional regulatory functions (82). Being rapidly raised in large amounts at the mucosal site, mucosal spike-specific IgA might serve as an early diagnosis biomarker, as already suggested (55, 56). More analyses on the fine epitope specificity of BAL IgA would be needed to improve the predictive value of BAL IgA in severe COVID-19 outcomes.

A mucosal vaccine targeting SARS-CoV-2 RBD administered *via* oral or nasal targets to induce secretion of IgA within the upper respiratory tract mucosa has been designed and tested (83, 84). In preclinical models, a vaccine-induced IgA was efficient at preventing COVID-19 development, but also at blocking viral transmission. Furthermore, nasal vaccination could not only be used for initial vaccination but also as a boost (85, 86). Hence, anti-spike/N IgA could also eliminate virally infected cells by ADCC (87) or ADCP (77) as shown in other mucosal viral diseases (14, 26, 60) using innate immune cells expressing Fcα-receptor and acting as second-chance protection.

In conclusion, this study highlights the similarities and differences between systemic and mucosal host immune responses after SARS-CoV-2 infection. Our findings revealed that sustained levels of S1-, RBD-, S2-, and NP-specific IgG and S1-specific IgA once SARS-CoV-2 was cleared from the lungs were associated with fatal outcomes. The loss of neutralizing activity in non-survivors at later stages of COVID-19 suggested that the persisting antibodies might be non-neutralizing although preserving functions mediated by Fc-R expressing myeloid cells. Further studies are needed to understand the role of non-protective antibodies in the pathogenesis of fatal COVID-19 disease, especially the interaction with innate immune cells *via* FcαR and their involvement in the cytokine storm. These findings are relevant to the design of new strategies for generating effective sterilizing vaccines and therapeutics, especially in COVID-19 convalescent individuals.

## Data availability statement

The original contributions presented in the study are included in the article/**Supplementary Material**. Further inquiries can be directed to the corresponding author.

## Ethics statement

The studies involving human participants were reviewed and approved by ethical committee for research (CER) of the University of Paris Saclay (CER-Paris-Saclay-2020- 050). The patients/participants provided their written informed consent to participate in this study.

## Author contributions

MR, GS, AC-C, DT, FR, AZ, CP, CC, NgT, DC, LL, and MB designed, performed, and analyzed the experiments. HL and NcT, performed the statistical analyses. AR, J-DC, GG DA, PM, and ECB provided the samples and analyzed clinical patient data. MR, MB, LL, GS, and NgT wrote the MS. All authors validated the study. MB led the study. All authors contributed to the article and approved the submitted version.

## Funding

This study was funded by joint fundings by the Agence Nationale de la Recherche (France) and Fondation pour la Recherche Médicale (FRM, France): Flash COVID ANR-FRM: ANR-20-COVI-0024 to MB and LL and the Line Renaud-Loulou Gasté fund to MB. The funders of the study had no role in study design, data collection, data analysis, data interpretation, or writing of the manuscript. AZ was supported by the China Scholarship Council; JR and AC-C were supported by the FRM.

## Acknowledgments

The authors greatly acknowledge Karine Bailly and Muriel Andrieu of the Cochin Cytometry and Immunobiology Facility for cytokine analyses during the COVID-19 confinement period.

## Conflict of interest

The authors declare that the research was conducted in the absence of any commercial or financial relationships that could be construed as a potential conflict of interest.

## Publisher’s note

All claims expressed in this article are solely those of the authors and do not necessarily represent those of their affiliated organizations, or those of the publisher, the editors and the reviewers. Any product that may be evaluated in this article, or claim that may be made by its manufacturer, is not guaranteed or endorsed by the publisher.
