# Supplementary material for: Persistent but dysfunctional mucosal SARS-CoV-2-specific IgA and low lung IL-1β associate with COVID-19 fatal outcome: A cross-sectional analysis
Source: Front Immunol. 2022 Sep 29;13:842468. doi: 10.3389/fimmu.2022.842468 (PMC9560774; doi:10.3389/fimmu.2022.842468)
Supplement: Supplementary file 1 [file DataSheet_1.pdf]

Persistent but Dysfunctional Mucosal SARS-CoV-2-specific IgA and Low Lung IL-1beta associate with COVID 19 Fatal Outcome: a Cross-sectional Analysis

Maria Julia Ruiz [1,2,3], Gabriel Siracusano[4], Andréa Cottignies-Calamarte [1,2,3], Daniela Tudor [1,2,3], Fernando Real [1,2,3], Aiwei Zhu [1,2,3,] Claudia Pastori [4], Claude Capron [5], Arielle R. Rosenberg [1,2,3,7], Nigel Temperton [6], Diego Cantoni [6], Hanqing Liao [8], Nicola Ternette [8], Pierre Moine [9], Mathieu Godement [9], Guillaume Geri [10,11], Jean-Daniel Chiche [12], Djillali Annane [9],

Supplementary Figures and Legends

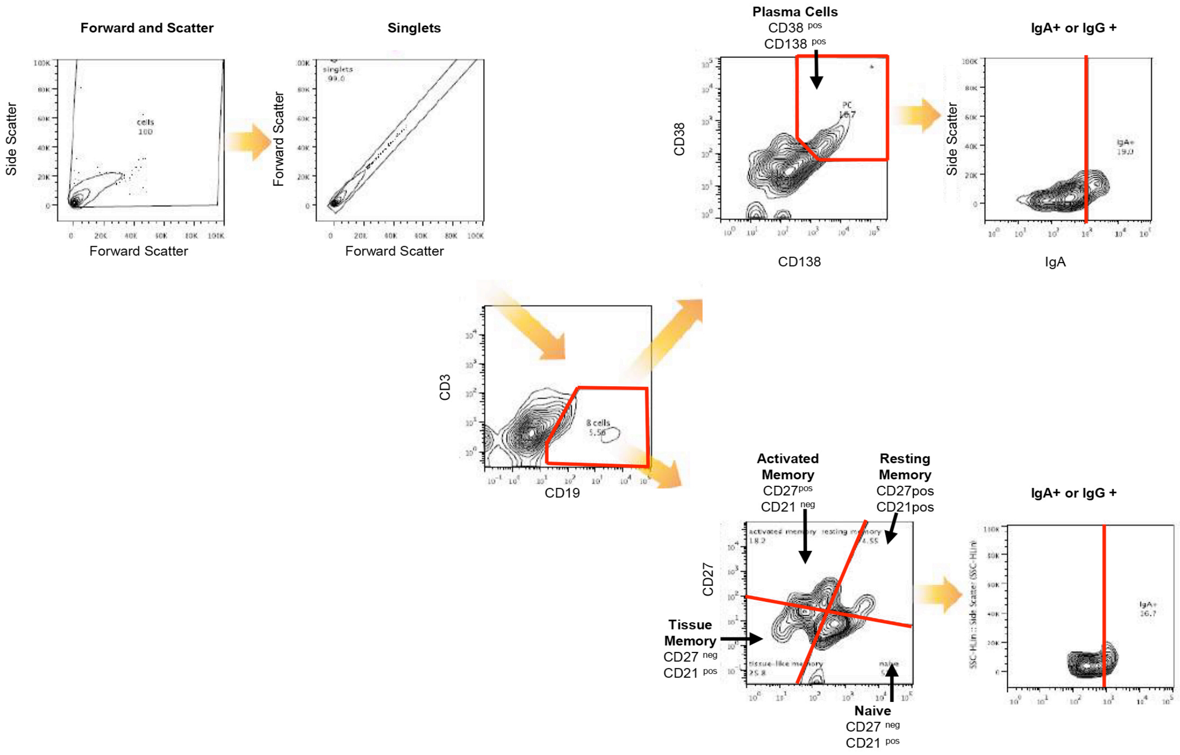

S1: Flow cytometry gating strategy for the characterization of B cell from BAL of SARS-CoV-2 **infected individuals.** Representative dot plot of BAL single cell suspensions studied by multiparametric flow cytometry, showing the gating strategy. After cell-doublet exclusion (Forward Scatter *vs* Forward Scatter, Singlet Dot Plot) B cells were gated based on their expression of CD19. After gating on CD19+ B cell population, Naïve B cells were defined as CD27^{neg} CD21^{pos}, Resting Memory B cells as CD27^{pos} CD21^{pos}, Activated Memory B cells as

CD27pos CD21neg, Tissue Memory B cells as CD27neg CD21pos and Plasma B cells as CD38pos CD138pos. Each population was then gated to measure their expression of IgG or IgA.

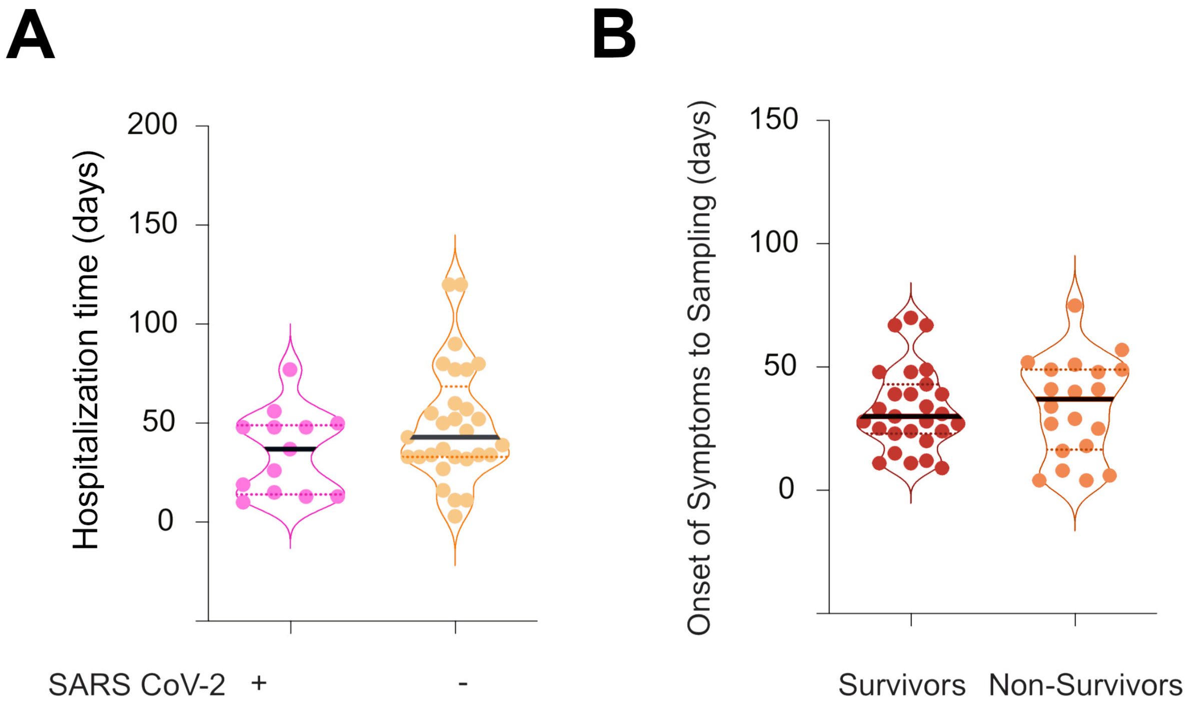

S2: Hospitalization time in SARS-CoV-2 **infected individuals. (A)** Comparison of the duration of hospitalization (days) in SARS-CoV-2+ BAL and SARS-CoV-2- BAL individuals. **(B)** Comparison of the time of onset of symptoms to sampling in Survivors *vs* Non-Survivors.

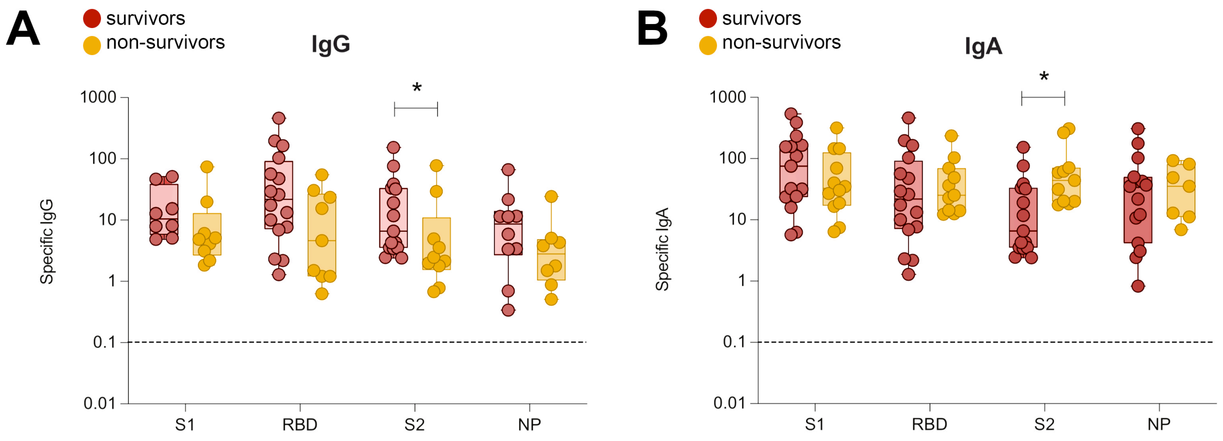

S3: Comparison of specific IgG and IgA anti SARS-CoV-2 antibody response in Survivors *vs* Non-Survivors. (A) Comparison between IgG responses against S1, S2, RBD and NP in Survivors vs Non-Survivors. **(B)** Comparison between IgA responses against S1, S2, RBD and NP in Survivors vs Non-Survivors. *p* values were calculated by using *Mann-Whitney* test. Dashed line: Cut-off value for detection.
